# Down-regulation of NTPDase2 and ADP-sensitive P2 Purinoceptors Correlate with Severity of Symptoms during Experimental Autoimmune Encephalomyelitis

**DOI:** 10.3389/fncel.2017.00333

**Published:** 2017-10-30

**Authors:** Marija Jakovljevic, Irena Lavrnja, Iva Bozic, Danijela Savic, Ivana Bjelobaba, Sanja Pekovic, Jean Sévigny, Nadezda Nedeljkovic, Danijela Laketa

**Affiliations:** ^1^Institute for Biological Research Sinisa Stankovic, University of Belgrade, Belgrade, Serbia; ^2^Département de Microbiologie-Infectiologie et d’Immunologie, Faculté de Médecine, Université Laval, Québec, QC, Canada; ^3^Centre de Recherche du CHU de Québec, Université Laval, Québec, QC, Canada; ^4^Institute for Physiology and Biochemistry, Department for General Physiology and Biophysics, Faculty of Biology, University of Belgrade, Belgrade, Serbia

**Keywords:** ectonucleoside triphosphate diphosphohydrolase-2 (NTPDase2), ADP-sensitive P2 receptors, experimental autoimmune encephalomyelitis (EAE), CD4^+^ T cells, neuroinflammation

## Abstract

The present study explores tissue and cellular distribution of ectonucleoside triphosphate diphosphohydrolase 2 (NTPDase2) and the gene and protein expression in rat spinal cord during the course of experimental autoimmune encephalomyelitis (EAE). Given that NTPDase2 hydrolyzes ATP with a transient accumulation of ADP, the expression of ADP-sensitive P2 purinoceptors was analyzed as well. The autoimmune disease was actively induced in Dark Agouti female rats and the changes were analyzed 10, 15 and 29 days after the induction. These selected time points correspond to the onset (***Eo***), peak (***Ep***) and recovery (***Er***) from EAE. In control animals, NTPDase2 was confined in the white matter, in most of the glial fibrillary acidic protein (GFAP)-immunoreactive (*ir*) astrocytes and in a considerable number of nestin-*ir* cells, while the other cell types were immunonegative. Immunoreactivity corresponding to NTPDase2 decreased significantly at ***Eo*** and ***Ep*** and then returned to the baseline levels at ***Er***. The preservation of the proportion of GFAP single-labeled and GFAP/NTPDase2 double-labeled elements along the course of EAE indicated that changes in NTPDase2-*ir* occurred at fibrous astrocytes that typically express NTPDase2 in normal conditions. Significant downregulation of P2Y_1_ and P2Y_12_ receptor proteins at ***Eo*** and several-fold induction of P2Y_12_ and P2Y_13_ receptor proteins at ***Ep*** and/or ***Er*** were observed implying that the pathophysiological process in EAE may be linked to ADP signaling. Cell-surface expression of NTPDase2, NTPDase1/CD39 and ecto-5′-nucleotidase (eN/CD73) was analyzed in CD4^+^ T cells of a draining lymph node by fluorescence-activated cell sorting. The induction of EAE was associated with a transient decrease in a number of CD4^+^ NTPDase2^+^ T cells in a draining lymph node, whereas the recovery was characterized by an increase in NTPDase2^+^ cells in both CD4^+^ and CD4^−^ cell populations. The opposite was found for NTPDase1/CD39^+^ and eN/CD73^+^ cells, which slightly increased in number with progression of the disease, particularly in CD4^−^ cells, and then decreased in the recovery. Finally, CD4^+^ NTPDase2^+^ cells were never observed in the spinal cord parenchyma. Taken together, our results suggest that the process of neuroinflammation in EAE may be associated with altered ADP signaling.

## Introduction

Multiple sclerosis (MS) is a chronic autoimmune disease characterized by isolated plaques of demyelination and axonal loss in the brain and spinal cord (Lassmann et al., 2007; Lassmann, 2014). Although the exact cause of MS is still undetermined, the disease is mediated by adaptive immunity through the infiltration of T cells into the central nervous system (CNS; for review see, Bjelobaba et al., 2017). Clinical studies in MS and studies on a widely accepted animal model of MS, experimental autoimmune encephalomyelitis (EAE), demonstrate that the autoimmune process is primarily mediated by self-reactive Th1 and Th17 CD4^+^ cells which enter the CNS, where they become reactivated by resident antigen-presenting microglia, recurrently inducing microglial activation and consequent demyelination and axonal loss (McFarland and Martin, 2007).

Besides mononuclear infiltrates and activated microglia, recent data imply that astrocytes are another key player in the pathophysiology of MS/EAE (Brück, 2005; Voskuhl et al., 2009; Lavrnja et al., 2015). In spite of the large heterogeneity, two main types of astrocytes have been recognized in the mammalian CNS, based on their morphology and tissue distribution (for review see, Oberheim et al., 2012); protoplasmic astrocytes with radially oriented processes are present in the gray matter in a non-overlapping domain arrangement, whereas fibrous astrocytes, which extend numerous overlapping processes along fiber tracts populate the white matter (Oberheim et al., 2008). Both types of astrocyte support neuronal stability and contribute to the maintenance of blood–brain barrier and general homeostasis (for review, see Simard and Nedergaard, 2004). In neuroinflammatory conditions associated with MS/EAE, astrocytes acquire activated phenotype and exert both proinflammatory and neuroprotective actions along the course of the disease (for review, see Pekny et al., 2016; Bjelobaba et al., 2017).

Therefore, for MS/EAE to develop, the coordinated action of several immune and neural cell types is required. One of the potent triggers of the neuroinflammatory cascade is ATP, which is released in response to diverse noxious stimuli in the brain (Melani et al., 2005; Rodrigues et al., 2015). When present in high concentrations, extracellular ATP acts as a danger-associated molecular pattern and its signaling through multiple P2 purinoceptors alarms the immune system (Di Virgilio et al., 2009; Fiebich et al., 2014). ATP initiates and coordinates a cross-talk between infiltrated T-cells and resident microglia and astrocytes (Liu et al., 2011), by recruiting T cells and facilitating their extravasation into the CNS (Mills et al., 2012), potentiating the release of cytokines and chemokines (Bours et al., 2006) and activating and attracting microglia and astrocytes (Franke et al., 2012), which govern further pathology in MS (Brück, 2005). Moreover, ATP activates low-affinity P2X7 receptors and potentiates the release of interleukin-1β and cyclooxygenase induction (Morán-Jiménez and Matute, 2000), causing demyelination, oligodendrocyte death and axonal damage (for review, see Matute, 2008).

Extracellular ATP is eliminated by the coordinated action of ectonucleotidase enzyme cascade. Specifically, ectonucleoside triphosphate diphosphohydrolase-1 (NTPDase1/CD39) and ectonucleoside triphosphate diphosphohydrolase 3 (NTPDase3) hydrolyze ATP to ADP and ultimately to AMP, while ecto-5′-nucleotidase (eN/CD73) catalyzes the last step of dephosphorylation, which results in adenosine production (Zimmermann, 2000; Yegutkin, 2008). The uniqueness of this enzyme cascade is that each step generates a ligand for a distinct set of purinergic receptors. Namely, ATP activates a subset of P2X1-7 receptor channels (Khakh and North, 2012) and G-protein coupled P2Y_1–14_ receptors (Abbracchio et al., 2006), while adenosine acts at G-protein coupled P1 purinoceptors (Bours et al., 2006; Ciruela et al., 2006). Yet another ectonucleotidase, with specific substrate affinity and restricted expression in the brain is ectonucleoside triphosphate diphosphohydrolase-2 (NTPDase2; previously known as Ecto-ATPase, or CD39L1). In the rodent brain, NTPDase2 is localized at specialized astrocytes, such as laminar astrocytes associated with fiber tracts in the brain stem and cerebrum (Braun et al., 2003, 2004), satellite astrocytes in the dorsal root ganglion (Braun et al., 2004), tanycytes, non-stellate astrocytes in the gray matter of discrete regions, like habenula (Gampe et al., 2012) and astrocyte-like progenitor cells of the subventricular zone (SVZ) of the lateral ventricle (Shukla et al., 2005; Mishra et al., 2006; Gampe et al., 2015). The enzyme preferentially catalyzes the dephosphorylation of ATP to ADP, generating a ligand for P2Y_1_, P2Y_12_ and P2Y_13_ receptors (Abbracchio et al., 2006; Burnstock, 2007). There is compelling evidence for the critical involvement of purinergic signaling in the control of different aspects of MS/EAE pathophysiology. A decreased plasma level of adenosine, reduced number of regulatory CD39^+^ T cells (Treg) and the reduced expression of A1 receptor in lymphocytes, together with the dysfunction of A1, A2A and P2X_7_ receptors and their altered expression in the proximity of demyelination lesions in the brain, are molecular signatures of MS (for review, see Cieślak et al., 2011; Burnstock, 2016). Furthermore, pharmacological blockade of A1 and A2A receptors results in beneficial therapeutic effects and reduced excitotoxicity and demyelination in MS (Antonioli et al., 2013; Safarzadeh et al., 2016). Other studies demonstrated important roles for CD4^+^CD73^+^ and CD4^+^CD39^+^ Th17 cells in the induction (Mills et al., 2008, 2012) and progression of EAE (Hernandez-Mir and McGeachy, 2017). These studies revealed close coupling between purinergic signaling and the presentation and severity of disease (Lavrnja et al., 2015). More recent data show that the ADP-sensitive P2 receptors are involved in the pathophysiology of MS/EAE (Amadio et al., 2010, 2014; Qin et al., 2017; Zhang et al., 2017). Specifically, a decrease in P2Y_12_ receptor protein expression in the proximity of MS lesions directly correlates with the extent of demyelination (Amadio et al., 2010) and the severity of disease (Zhang et al., 2017). Since ADP is generated by the catalytic action of NTPDase2, we hypothesized that the enzyme may have a role in neuroinflammatory processes during MS/EAE, through the modulation of P2Y-mediated signaling and cell communication. For this reason, we have analyzed the expression and cellular distribution of NTPDase2 in the lumbosacral spinal cord of Dark Agouti rats (DA) during EAE.

## Materials and Methods

### Animals

Eight-week-old female rats of DA strain from the local colony were used in the study. Littermates were kept in the same cage (4–5/cage) under constant temperature and humidity, 12 h light/dark cycle and a free access to laboratory chow and tap water. Experimental protocols were approved by the Ethical Committee for the Use of Laboratory Animals of the Institute for Biological Research “Sinisa Stankovic”, University of Belgrade, Serbia (No. 01-11/14) and were in compliance with the EEC Directive (2010/63/EU) on the protection of animals used for experimental and other scientific purposes.

### Induction of Experimental Autoimmune Encephalomyelitis (EAE)

Animals selected for the study were randomly divided into two groups. In the first group, the animals under ether anesthesia received a subcutaneous injection of an encephalitogenic emulsion in the hind footpad. The emulsion was prepared by mixing equal volumes of rat spinal tissue homogenate (50% *w/v* in saline), and complete Freund’s adjuvant (0.5 mg/mL *Mycobacterium tuberculosis*; Sigma, St. Louis, MO, USA). All immunized animals developed signs of acute EAE. A group of age-matched naïve female rats was used as control group.

**Figure 1:**
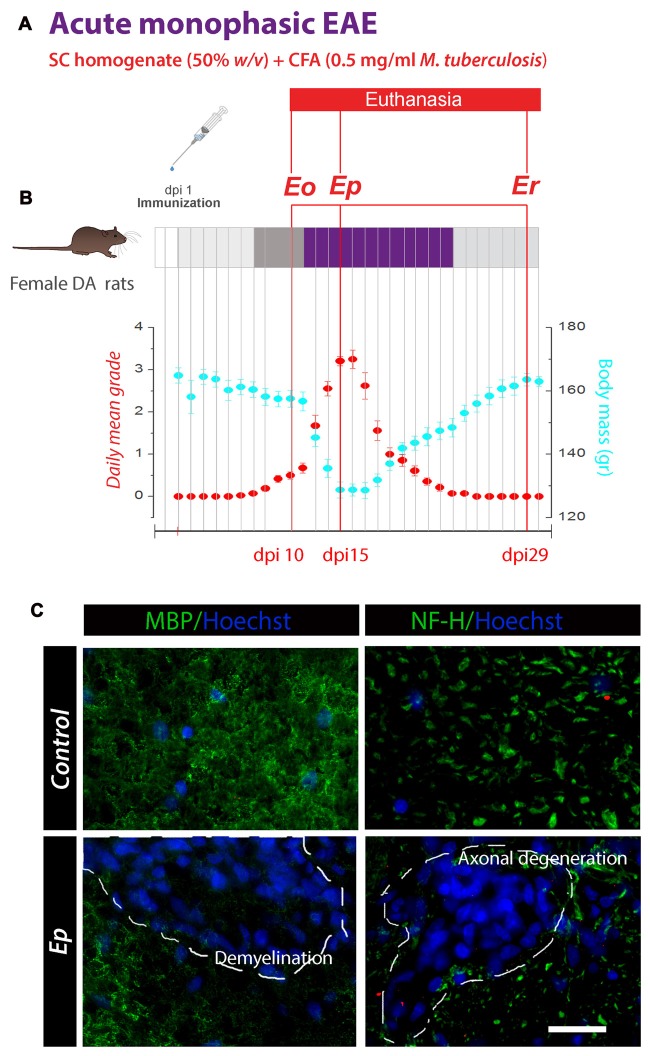
Schematic representation of experimental protocol used for the induction of monophasic experimental autoimmune encephalomyelitis (EAE) in Dark Agouti (DA) rats. **(A)** Animals immunized on day 1 (dpi 1) by *s.c.* injection of the encephalitogenic emulsion developed a disease with 100% incidence. **(B)** Animals were weighed and scored daily for neurological signs of EAE using standard 0–5 EAE grading scale. Plots represent the variations in daily mean disease grade ± SEM (*red circles*) and mean body mass (*blue circles*) during EAE, measured in two separate rounds of EAE inductions. The animals were euthanized at 10, 15 and 29 days after immunization, i.e., at the time-points which correspond to the onset of disease ***(Eo)***, the peak of neurological symptoms ***(Ep)*** and recovery ***(Er)***. **(C)** Representative micrographs showing the autoimmune-mediated demyelination and axonal degeneration in the spinal cord ventral white matter. Double immunofluorescence (IF) labeling was performed using antibodies directed against myelin basic protein (MBP; left—*green fluorescence*) or heavy neurofilament subunit, NF-H (right—*green fluorescence*). Nuclei were counterstained with Hoechst (*blue fluorescence*). Dotted areas show patches of the neurodegeneration. Scale bar at **C** = 20 μm.

Animals were weighed and monitored for the neurological signs of the disease every day (Figure 1A). During a 30-day post-induction period (dpi), two unbiased examiners evaluated the severity of the disease, using standard EAE grading scale (Milicevic et al., 2003), which converts symptoms to grades, as follows: unaffected—0; reduced tail tone—0.5; tail atony—1; slightly/moderately clumsy gait, impaired righting ability or combination—1.5; hind limb paresis—2; partial hind limb paralysis—2.5; complete hind limb paralysis—3; complete hind limb paralysis with forelimb weakness—3.5; tetraplegic—4 and moribund state or death—5. The daily mean grade was calculated by averaging daily grades of the animals within the group. The study included two separate EAE induction and sample preparation.

The immunization resulted in the acute monophasic disease, with 100% incidence and recovery. The first signs of EAE in individual animals appeared at 9 dpi, while at 10 dpi all animals displayed reduced or lost tail tone. The symptoms progressively advanced during the next 5 days, with the most severe presentation of the disease observed between 14 and 15 dpi. After this point, animals began to recover and complete remission was observed at 24 dpi. Given the monophasic course of the disease, animals were selected and euthanized at the three time-points after the immunization, i.e., at 10, 15 or 29 dpi, which corresponded to the onset (***Eo***), the peak (***Ep***) and the recovery (***Er***) phase of the disease (Figure 1B).

### Spinal Cord Dissection and Tissue Preparation

The animals were anesthetized and perfused with 0.9% sodium chloride, positioned in Harvard apparatus and decapitated. Spinal cords were dissected on ice and their lumbosacral parts isolated for crude plasma membrane preparation, RNA extraction and cryosectioning.

### Crude Plasma Membrane Preparation

The dissected lumbosacral parts of the spinal cords were pooled for each group (3/group) and used for crude plasma membrane preparation by following the protocol of Gray and Whittaker (1962). Briefly, the tissue was homogenized in the isolation buffer (0.32 mol/L sucrose, 5 mmol/L Tris, pH 7.4) and centrifuged at 3000× *g* for 10 min at 4°C. Supernatant was separated and centrifuged again at 12,000× *g* for 40 min at 4°C. The resulting pellets were combined and resuspended in ice-cold 5 mmol/L Tris pH 7.4. The protein content was determined using Micro BCA Protein Assay Kit (Thermo Fisher Scientific, Rockford, IL, USA).

### Western Blot

**Figure 2:**
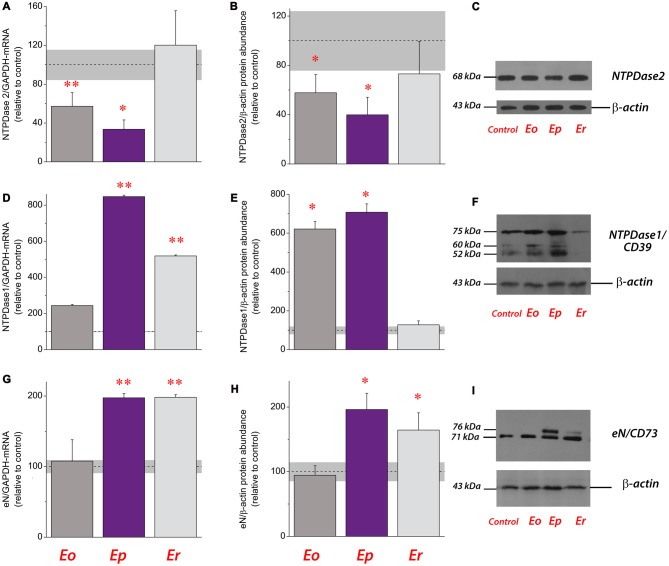
Expression analysis of ectonucleotidases during EAE. Transcriptional expression of ectonucleoside triphosphate diphosphohydrolase 2 (NTPDase2) **(A)**, NTPDase1/CD39 **(D)** and ecto-5′-nucleotidase (eN/CD73) **(G)**. Bars represent mean target/GAPDH-mRNA abundance (± SEM), determined in total RNA isolated from the lumbar spinal cord of control rats (100%) and rats at ***Eo***, ***Ep*** and ***Er***. Dot line represents mean target/GAPDH-mRNA abundance (± SEM, *gray area*) determined in control animals. Samples are from three animals per group. *p < 0.01; **p < 0.001 (analysis of variance, ANOVA followed by Tukey’s *post hoc* test). Relative protein abundance of NTPDase2 **(B)**, NTPDase1/CD39 **(E)** and eN/CD73 **(H)** in crude plasma membrane preparations obtained from control animals and at each phase of EAE. The intensity of each protein band of interest was assessed by densitometric measurements using ImageJ software and expressed relative to the optical density (OD) of the β-actin band in the same lane (target protein/β-actin ratio). The target protein/β-actin value obtained for the control sample was defined as 100% (*dot line*) ± SEM (*gray area*) and the ratios obtained for other samples were expressed relative to the control (*bars*). Bars represent the mean target protein abundance (± SEM) from *n* ≥ 4 determinations performed on at least two independent sample preparations. Significance inside the graph: *p < 0.01; **p < 0.001. Representative support membranes showing the position of bands corresponding to NTPDase2 **(C)**, NTPDase1/CD39 **(F)** and eN/CD73 **(I)**, visualized on X-Ray films with the use of chemiluminescence.

**Figure 3:**
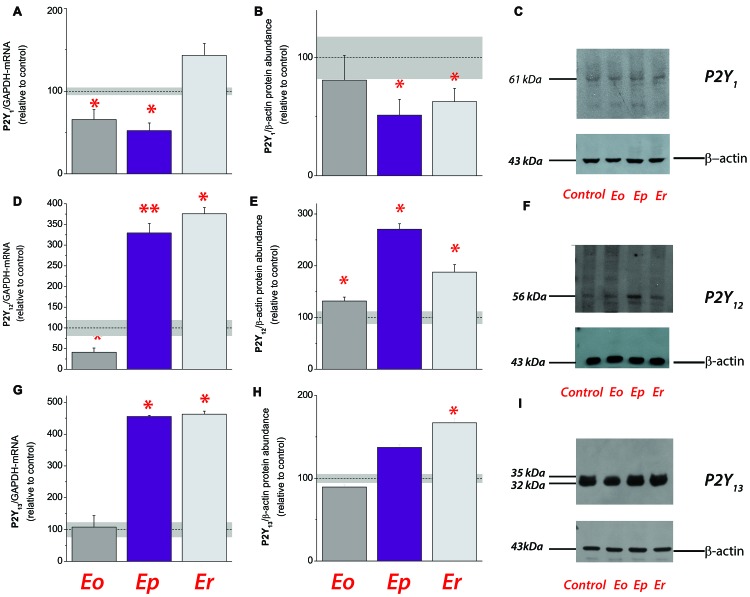
Expression analysis of ADP-sensitive purinoceptors during EAE. Transcriptional expression of P2Y_1_ **(A)**, P2Y_12_ **(D)** and P2Y_13_ **(G)**. Bars represent mean target/GAPDH-mRNA abundance (± SEM), determined in total RNA isolated from lumbar spinal cords of control rats (100%) and rats at ***Eo***, ***Ep*** and ***Er***. Dot line represents mean target/GAPDH-mRNA abundance ± SEM (*gray area*) determined in control animals. Samples are from three animals per each group. *p < 0.01 (ANOVA followed by Tukey’s *post hoc* test). Relative protein abundance of P2Y_1_ **(B)**, P2Y_12_ **(E)** and P2Y_13_ **(H)** in crude plasma membrane preparations obtained from control and EAE animals. The intensity of each specific band was assessed by densitometric measurements using ImageJ software and expressed relative to the OD of the β-actin band in the same lane (target band/β-actin ratio). The ratio obtained from control crude plasma membrane preparation was defined as 100% (*dot line*) ± SEM (*gray area*) and the ratios obtained for other samples were expressed relative to control (*bars*). Bars represent mean target-protein abundance (± SEM) from *n* ≥ 3 determinations performed on at least two independent sample preparation. Significance inside the graph: *p < 0.05, **p < 0.001. Representative support membranes showing the position of bands corresponding to P2Y_1_ **(C)**, P2Y_12_ **(F)** and P2Y_13_ **(I)**, visualized on X-Ray films with the use of chemiluminescence.

Membrane samples were diluted in 4× Laemmli sample buffer to a protein concentration of 1 mg/ml and incubated for 5 min at 95°C. Sample proteins (15 μg) were resolved by 7.5% PAGE-SDS gel under non-reducing conditions, and transferred to a support membrane (Immobilon-P transfer membrane, Millipore). For P2Y receptor protein detection, sample proteins were resolved under reducing conditions. The support membranes were blocked in 5% BSA or 5% Blotto (Santa Cruz Biotechnology) in Tris-buffer saline Tween-20 (TBST) and probed with primary antibodies (as indicated in Table 1), at 4°C overnight. After rinsing in TBST (3 × 5 min), the support membranes were incubated in the appropriate HRP-conjugated IgG secondary antibodies, for 2 h at room temperature. Bands were visualized on X-ray films (Kodak, Rochester, NY, USA) with the use of chemiluminescence (ECL, GE Healthcare). The amount of sample and titer of the antibodies were chosen to ensure optimum immunoassay potency (Supplementary Figure S1). Relative molecular weight of each band on the support membrane was estimated by the relative migration method and the bands of interest were chosen based on expected size(s). For NTPDase1/CD39 and eN/CD73, which exist in distinct molecular weight glycoforms, and P2Y13, which exists in two protein isoforms, multiple bands based on the expected size were integrated for a densitometric analysis (*ImageJ* software package). The positions of the specific bands are indicated in Figures 2, 3. The value obtained for a band of interest is normalized to the optical density (OD) of β-actin band on the same lane and presented relative to control (100%) ± SEM. The bars represent means from *n* ≥ 3 separate determinations. A list of primary and secondary antibodies is given in Table 1.

**Table 1:** List of antibodies.

Antibody selectivity	Source and type	Dilution	Manufacturer
NTPDase2 (rN2-6L)	Rabbit, *pc*	1:2000 (WB) 1:200 (IHC, IF) 1:100 (FC)	ectonucleotidases-ab.com;Cat# NTPDase2, RRID:AB_2314986 (Sévigny et al., 2002)
NTPDase1/CD39 (mN1-2C(I4,I5))	Guinea pig, *pc*	1:6000 (WB) 1:400 (FACS)	ectonucleotidases-ab.com;Cat# NTPDase1
ecto-5′-nucleotidase/CD73	Rabbit, *pc*	1:5000 (WB)	Cell Signaling Technology #3160, RRID:AB_11217629
ecto-5′-nucleotidase/CD73 (rNu-9L(I4,I5))	Rabbit, *pc*	1:100 (FACS)	ectonucleotidases-ab.com;Cat# ecto-5′-nucleotidase/CD73
P2Y_1_	Rabbit, *pc*	1:1000 (WB)	Alomone Labs; #APR-0009, RRID:AB_2040070
P2Y_12_	Rabbit, *pc*	1:1000 (WB)	Sigma P4817, RRID:AB_261954
P2Y_13_	Rabbit, *pc*	1:1000 (WB)	Alomone Labs; #APR-017, RRID:AB_2040076
GFAP	Mouse, *mc*	1:500 (IF)	UC Davis/NIH NeuroMab Facility (73–240), RRID:AB_10672298
Nestin	Mouse, *mc*	1:100 (IF)	Sigma N5413, RRID:AB_1841032
Iba1	Goat, *pc*	1:400 (IF)	Abcam ab5076, RRID:AB_2224402
ED1/CD68	Mouse, *mc*	1:100 (IF)	Abcam ab31630, RRID:AB_1141557
CD4	Mouse, *mc*	1:500 (IF) 1:100 (FACS)	Sigma-Aldrich SAB4700733, RRID:AB_476825733
NF-H (SMI32)	Mouse, *mc*	1:2000 (IF)	Covance Research Products Inc AB_509997, RRID:AB_509997
MBP	Mouse, *mc*	1:100 (IF)	BioLegend 801703, RRID:AB_510039
NG2	Mouse, *mc*	1:50 (IF)	Sigma N8912, RRID:AB_609907
Oligodendrocyte marker O4	Mouse, *mc*	1:100 (IF)	Millipore MAB345, RRID:AB_11213138
β-Actin	Mouse, *mc*	1:7500 (WB)	Sigma A5316, RRID:AB_476743
Anti-rabbit HRP conjugated IgG	Donkey, *pc*	1:5000 (IHC)	Santa Cruz, sc2305, RRID:AB_641180
Anti-rabbit IgG AlexaFluor 555	Donkey, *pc*	1:200 (IF)	Invitrogen A-21428, RRID:AB_141784
Anti-mouse IgG AlexaFluor 488	Donkey, *pc*	1:200 (IF, FACS)	Invitrogen A21202, RRID:AB_141607
Anti-mouse HRP conjugated IgG	Donkey, *pc*	1:5000 (WB)	Santa Cruz sc-2314, RRID:AB_641170
Anti-goat IgG Alexa Fluor 488	Donkey, *pc*	1:250 (IF)	Invitrogen A-11055, RRID:AB_142672
Anti-rabbit IgG AlexaFluor 488	Donkey, *pc*	1:200 (IF)	Invitrogen A-21206, RRID:AB_141708
Anti-mouse IgG AlexaFluor 555	Donkey, *pc*	1:200 (IF, FACS)	ThermoFisher Scientific A-31570, RRID:AB_2536180

*WB, Western blot; IFC, Immunohostochemistry; IF, immunofluorescence; FC, flow cytometry; mc, monoclonal; pc, polyclonal*.

### Immunohistochemistry for Light Microscopy and Immunofluorescence

Cross-cryosections of the lumbosacral spinal cord were used for immunohistochemical staining. For light microscopy, the sections were incubated in 1% hydrogen peroxide (in methanol) followed by incubation in 10% normal donkey serum, to block endogenous peroxidase and prevent non-specific labeling. The sections were probed with anti-NTPDase2 primary antibodies, overnight at 4°C, rinsed in PBS and incubated with secondary HRP-conjugated donkey anti-rabbit antibodies, for 2 h, at room temperature. The identity of the antibodies and dilutions used for immunohistochemistry are indicated in Table 1. The signal was visualized using 3,3′-S-diaminobenzidine-tetrahydrochloride (DAB, Dako, Glostrup, Denmark) as a substrate. Incubation without primary antibodies resulted in the absence of any specific reaction. After dehydration, sections were mounted using DPX Mounting medium (Fluka, Buchs, Switzerland), and examined under a Zeiss Axiovert microscope (Carl Zeiss GmbH, Vienna, Austria).

For double immunofluorescence (IF) labeling, 20-μm thick cryosections blocked with 1% BSA (Thermo Fisher Scientific, Rockford, IL, USA) were incubated with anti-NTPDase2 antibodies, overnight at 4°C. After thorough rinsing in PBS, secondary donkey anti-rabbit IgG Alexa Fluor 555 antibodies were applied. For second labeling, primary antibodies directed against glial fibrillary acidic protein (GFAP), ionizing calcium-binding adaptor molecule 1 (Iba1), CD68, nestin, neurofilament heavy polypeptide (NF-H), myelin basic protein (MBP) and oligodendrocyte marker 4 (O4), (overnight at 4°C) and secondary IgG AlexaFluor 488 antibodies were applied. Nuclei were counterstained by Hoechst 33342 dye (5 μg/ml—Life Technologies, Invitrogen, Carlsbad, CA, USA). Sections were mounted in Mowiol (Calbiochem, Millipore, Germany) and captured by a camera associated with Zeiss Axiovert fluorescent microscope (Zeiss, Jena, Germany).

### Quantification

The spinal cord sections from the lumbosacral segments, labeled for NTPDase2/GFAP or NTPDase2/nestin, were used for the quantification. Each section was arbitrarily divided into four quadrants, on the basis of the dorsal/ventral and right/left symmetry. Two to four microscopic fields per each quadrant were captured and stored as digital data. Digital images were examined and each quadrant was independently counted for single-labeled (NTPDase2, GFAP, nestin) and double-labeled (NTPDase2/GFAP and NTPDase2/nestin) cellular processes. In all cases, labeling in the respective single channels was directly compared with the superimposed images. Representative single-channel images of sections labeled for NTPDase2 and individual cell markers are presented in Supplementary Figures S2–S4. The results are expressed as mean % of single-labeled or double-labeled elements relative to the total number of labeled elements (±SEM), from six cross-sections of the spinal cord per animal (three animals per each EAE group).

### Quantitative Real-time PCR

Lumbosacral parts of the spinal cords (5/group) were frozen in liquid nitrogen and kept at −80°C until use. RNA extraction was performed using TRIzol^®^ reagent, according to the manufacturer’s instructions. RNA content was measured spectrophotometrically by determining OD at 260 nm, while the purity of the samples was assessed by determining OD_260_/OD_280_ and OD_260_/OD_230_ ratios. A volume equivalent to 1 μg of RNA was used for reverse transcription with High Capacity cDNA Reverse transcription kit (Applied Biosystems, Foster City, CA, USA). The obtained cDNA samples were used for real-time PCR analysis with the QuantStudio^TM^ 3 Real-Time PCR System (Applied Biosystems, Foster City, CA, USA). Reactions were carried out in a mixture consisting of 2 μl cDNA, 2 μl RNase-free water (UltraPure, Invitrogen, Germany), 5 μl QTM SYBR Green PCR Master Mix (Applied Biosystems, Foster City, CA, USA) and 0.5 μl primers (100 pmol/μl), under the following conditions: 10 min of enzyme activation at 95°C, 40 cycles of 15 s denaturation at 95°C, 30 s annealing at 64°C, 30 s amplification at 72°C and 5 s fluorescence measurement at 72°C. Primer sequences are listed in Table 2. Relative abundance of specific mRNA was determined by the 2^−ΔCt^ method, using GAPDH as the internal standard. The results are expressed as target-mRNA/GAPDH-mRNA ratio (relative to the ratio obtained for control).

**Table 2:** Primer sequences.

Target gene	Forward	Reverse		
*Nt5e*	CAAATCTGCCTCTGGAAAGC	ACCTTCCAGAAGGACCCTGT
*Entpd1*	TCAAGGACCCGTGCTTTTAC	TCTGGTGGCACTGTTCGTAG
*Entpd2*	CCCTCATGACCTTCTTCCTG	CCAAGAGACCCGGTATAGCA
*P2ry1*	CTGGATCTTCGGGGATGTTA	CTGCCCAGAGACTTGAGAGG
*P2ry12*	CGAAACCAAGTCACTGAGAGA	CCAGGAATGGAGGTGGTGTTG
*P2ry13*	GGCATCAACCGTGAAGAAAT	TTGGCAATCACCGTGTAAAA
*P2rx1*	GTGTTTGGGATTCACTTTGATA	TCTGCTTGTAGTAGTGCCTCTT
*P2rx2*	AGAAGAGTGACTACCTCAAGCA	ACAGTTCCAGTTGATGATGACT
*P2rx3*	CCTCACCGACAAGGACATA	ACACCCAGCCGATCTTAAT
*P2rx4*	TCCTGATAAGACCAGCATTT	CAAGAGGGTGAAGTTTTCTG
*P2rx5*	CAAATCTCTACTGTCCCATCTT	TAGTAGTGTGGGTTGCATTTAG
*P2rx7*	TCGGAGAGAACTTTACAGAGG	TCGGAGAGAACTTTACAGAGG
*Adora1*	GTGATTTGGGCTGTGAAGGT	GAGCTCTGGGTGAGGATGAG
*Adora2a*	TGCAGAACGTCACCAACTTC	CAAAACAGGCGAAGAAGAGG
*Adora2b*	CGTCCCGCTCAGGTATAAAG	CCAGGAAAGGAGTCAGTCCA
*Adora3*	TTCTTGTTTGCCTTGTGCTG	AGGGTTCATCATGGAGTTCG
*Gapdh*	TGGACCTCATGGCCTACAT	GGATGGAATTGTGAGGGAGA

### Flow Cytometry

Popliteal lymph nodes were removed, homogenized through a nylon sieve and centrifuged at 700× *g* for 3 min. Pellets were resuspended in PBS and filtered through sterile 50 μm filters. Cells were counted and 10^6^ cells were centrifuged and resuspended in PBS with 10% normal rat serum with primary antibodies directed to CD4 and NTPDase2, NTPDase1/CD39 and eN/CD73 (Table 1), for 45 min at 4°C. Cells were washed twice with PBS and incubated with the appropriate secondary antibodies (listed in Table 1). After thorough washing in PBS, flow cytometric analysis was performed in CyFlow^®^ Space Partec (Partec GmbH, Munster, Germany). Data were analyzed using PartecFloMax^®^ (Partec GmbH, Munster, Germany) software.

### Data Analysis

Statistical analyses were performed in OriginPro8 SR0 software package (v8.0724, OriginLab Corporation, Northampton, MA, USA), using one-way analysis of variance (ANOVA) followed by Tukey’s *post hoc* test. The data are presented as mean ± SEM and considered statistically significant at *p* < 0.05.

## Results

### Disease Symptoms and Histopathological Changes during EAE

The experimental design of the study is shown in Figure 1A. Active immunization led to a monophasic disease with 100% incidence and recovery. Daily mean disease grade and body mass variations during a 30-day period after induction (dpi) are presented in Figure 1B. A maximum daily mean grade and a peak of body weight reduction were demonstrated at 15 dpi, which was defined as the peak of disease (***Ep***). To confirm that the neurological deficits in EAE animals were caused by autoimmune-mediated demyelination and axonal degeneration, IF labeling directed to MBP and heavy neurofilament subunit (NF-H) was performed (Figure 1C). Immunolabeling for MBP and NF-H revealed large areas of neurodegeneration in the spinal white matter at ***Ep***.

### Expression of NTPDase2 and ADP-Sensitive P2 Purinoceptors Change Over the Course of the Disease

The expression of NTPDase2 was analyzed in the lumbosacral part of the spinal cords during EAE. A strong down-regulation of NTPDase2 gene and protein expression at ***Eo*** and ***Ep*** and the baseline expression at ***Er*** were observed (Figure 2). At the peak of disease, the abundance of NTPDase2, at mRNA (Figure 2A) and protein (Figures 2B,C) levels decreased to about 30% (*p* < 0.001) and 40% (*p* < 0.001) of those detected in naïve animals, respectively. Since purine nucleotides are dephosphorylated in a stepwise manner by a coordinated action of ectonucleotidases, gene and protein expression profiles of two major ectonucleotidases, NTPDase1/CD39 and eN/CD73 were determined as well (Figure 3). A strong induction of NTPDase1/CD39 (Figures 2D–F) and eN/CD73 (Figures 2G–I) was demonstrated at mRNA (Figures 2D,G) and protein levels (Figures 2E,H) at ***Ep*** and ***Er***.

Together, the results obtained imply a general shift in the availability of ligands for P1 and P2 receptors during EAE. To estimate the physiological significance of the observed alterations and to gain a better insight into the circumstances in which they occurred, we analyzed the expression profiles of ADP-sensitive P2Y_1_, P2Y_12_ and P2Y_13_ receptors. The expression of P2Y_1_ (Figures 3A–C) and P2Y_12_ receptors (Figures 3D–F) consistently decreased at mRNA and protein levels at ***Eo***, whereas at ***Ep*** and/or ***Er***, P2Y_12_ (Figures 3D–F) and P2Y_13_ (Figures 3G–I) receptor gene and protein expression increased several-fold. We further screened the abundance of mRNA coding for ATP-sensitive P2X and adenosine P1 receptors during EAE, in order to evaluate the possible consequences on ATP- and adenosine-mediated signaling. A consistent decrease in the abundance of transcripts coding for P2X_1_, P2X_2_, P2X_3_, P2X_4_, P2X_5_ and P2X_7_ purinoceptors, particularly during the symptomatic stages of the disease, indicated the attenuation in P2X-mediated ATP signaling, while a significant decrease in A_1-_ and A_2B-_, and an up-regulation of A_2A_- and A_3_-mRNA levels, were observed (Table 3).

**Table 3:** Expression profiles for selected P2X and P1 receptor genes.

	Target gene/GAPDH-mRNA (% of control)		
Target gene	*Eo*	*Ep*	*Er*
P2X_1_	40.1 ± 17.5*	78.5 ± 22.6	78.1 ± 32.9
P2X_2_	66.8 ± 1.7	32.5 ± 0.5*	96.8 ± 21.1
P2X_3_	31.3 ± 7.1*	11.8 ± 0.6*	74.6 ± 16.0
P2X_4_	36.5 ± 26.9*	105.4 ± 9.8	161.0 ± 17.273
P2X_5_	75.1 ± 19.0	22.5 ± 15.1*	99.5 ± 26.1
P2X_7_	69.7 ± 14.6*	72.4 ± 11.6	182.9 ± 36.2
A_1_	65.6 ± 9.7*	40.3 ± 4.2*	98.9 ± 14.2
A_2A_	197.6 ± 93.7	125.0 ± 3.0	138.0 ± 15.3
A_2B_	55.1 ± 11.8*	68.5 ± 27.0*	134.3 ± 45.73
A_3_	366.4 ± 40.0*	329.5 ± 11.5*	299.2 ± 8.1*

Statistical significance: *p < 0.05.

**Figure 4:**
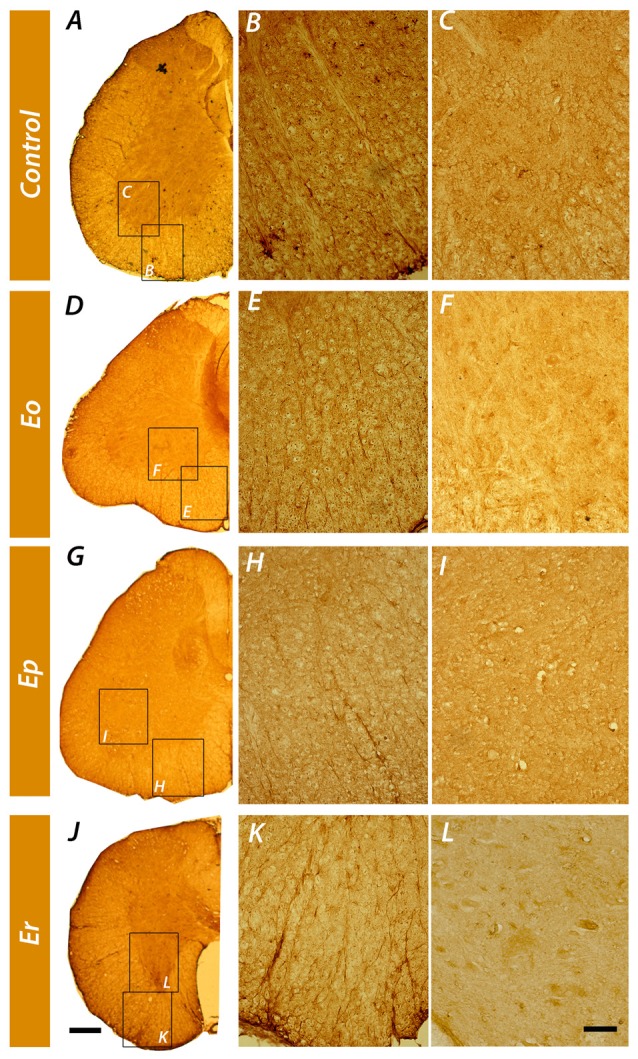
Immunohistochemical localization of NTPDase2 in the lumbosacral spinal cord. Low-power magnification micrographs showing the distribution of NTPDase2-*ir* elements in control spinal cord cross-sections **(A)** and during the course of the disease **(D,G,J)**. High-power micrographs of the areas enclosed by rectangles denoted with the same letter, show NTPDase2-*ir* in spinal cord white **(B,E,H,K)** and gray matter **(C,F,I,L)** in control sections and over the course of EAE. Scale bar at **(J)** applicable to **A,D,G** = 200 μm; Scale bar at **(L)**, applicable to **B,C,E,F,H,I,K** = 20 μm.

Tissue localization of NTPDase2 in the spinal cord was demonstrated by immunohistochemistry, using the well-characterized rN2-6_L_ antibodies directed to NTPDase2 (Figure 4). In control sections, strong NTPDase2 immunoreactivity (*ir*) was confined mainly to fibrous elements in the white matter (Figure 4B), whereas labeling in the gray matter was weak and indistinct (Figure 4C). Light microscopic examination implied that the NTPDase2-*ir* elements in the white matter corresponded to fibrous astrocytes processes. In sections obtained at ***Eo*** and ***Ep*** the overall intensity of NTPDase2-*ir* decreased (Figures 4E,H), while at ***Er***, the intensity of NTPDase2-*ir* was comparable to the intensity observed in control sections (Figure 4K).

### NTPDase2 Immunofluorescence

**Figure 5:**
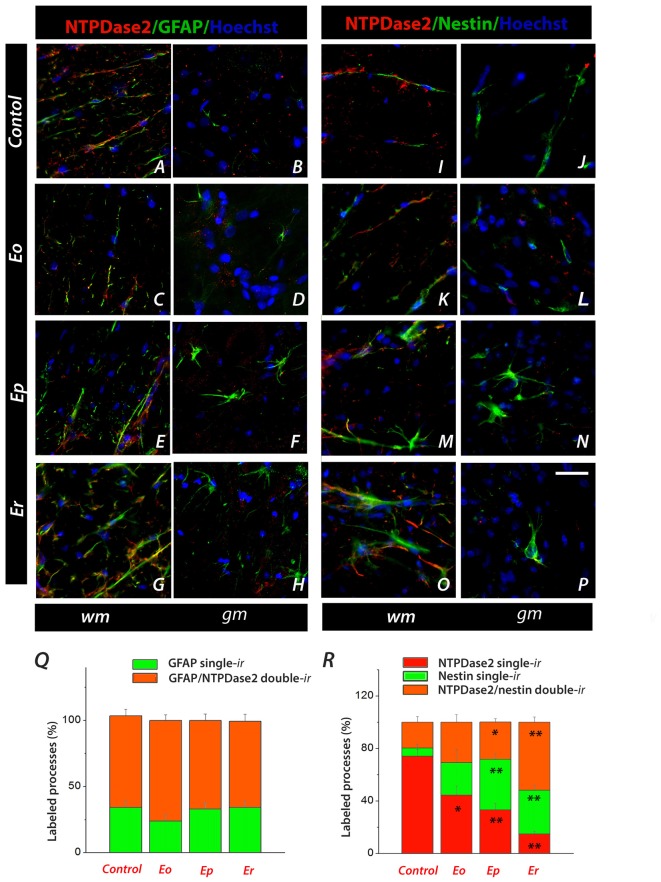
Characterization of NTPDase2-*ir* spinal cord cross-sections during EAE by using double IF labeling. **(A–H)** Representative micrographs showing colocalization of NTPDase2-*ir* (*red fluorescence*) and glial fibrillary acidic protein (GFAP)-*ir* (*green fluorescence*) in spinal white (*wm*) and gray (*gm*) matter, in a control section and during EAE. **(Q)** Quantification of GFAP single-labeled and GFAP/NTPDase2 double-labeled processes. Bars represent mean % of GFAP single-labeled (*green bars*) and GFAP/NTPDase2 double-labeled (*orange bars*) fibrous elements, relative to the total number of labeled processes (± SEM), counted from at least six sections, from three different animals per EAE phase. **(I–P)** Representative micrographs showing localization of NTPDase2 single-labeled (*red fluorescence*), nestin single-labeled (*green fluorescence*) and NTPDase2/nestin double-labeled elements (*orange)* in control sections and over the course of EAE. Nuclei are counterstained with Hoechst (*blue fluorescence*). The scale bar at **(P)** applicable to all micrographs = 20 μm. **(R)** Quantification of NTPDase2 single-labeled (*red bars*), nestin single-labeled (*green bars*) and NTPDase2/nestin double-labeled (*orange bars*) fibrous elements. Results present mean % of each label relative to a total number of labeled processes (± SEM), counted from at least six sections, from three different animals per each EAE phase. Significance inside of graph: *p < 0.05, **p < 0.001.

The precise cellular localization of NTPDase2 in the spinal cord was further determined by double IF labeling with antibodies directed to known cellular markers. Astrocytic localization of NTPDase2 was assessed by double IF labeling of NTPDase2 and GFAP (Figure 5, Supplementary Figure S2). In sections obtained from naïve animals, NTPDase2-*ir* was detected in the white matter only, in association with most of the GFAP-*ir* fibrous astrocytes (Figure 5A). During the course of EAE, the overall intensity of GFAP-*ir* progressively increased at hypertrophied astrocytes throughout the spinal cord parenchyma, while the intensity of NTPDase2-*ir* visibly decreased in the white matter (Figures 5C,E,G). However, the number of GFAP single-labeled and GFAP/NTPDase2 double-labeled processes determined at each phase of EAE revealed that, although the intensity of NTPDase2-*ir* varied during EAE, the number of double-labeled GFAP/NTPDase2 elements remained the same during the whole course of the disease, indicating that the observed changes in the NTPDase2 expression occurred in the same subset of cells.

In inflammatory conditions, astrocytes undergo significant morphological and functional changes and express many different molecules, including intermediate filament protein nestin, a marker of neural stem and progenitor cells. Therefore, we next tested whether the NTPDase2 labeled astrocytes displayed heterogeneity regarding the expression of nestin (Figures 5I–P, Supplementary Figure S3). In control conditions, nestin-*ir* was detected in fibrous elements (Figures 5I,J) and partially overlapped with GFAP-*ir* (data not shown). Among the immunolabeled processes in the white matter, about 75% and 5% were either NTPDase2- or nestin single-labeled elements, while the remaining 20% were double-labeled for NTPDase2 and nestin (Figure 5I). During the course of EAE, overall nestin-*ir* increased (Figures 5K–N). In the gray matter, nestin-*ir* was observed in oval (Figures 5L,N) and fusiform cells (Figure 5P) with multiple radial processes, which were never NTPDase2-*ir*. In the white matter, both the overall intensity and the number of nestin-*ir* processes increased (Figures 5K,M), resulting in about 40% of nestin single-labeled and about 30% of NTPDase2/nestin double-labeled elements at ***Ep*** (Figure 5R). In the final phase of EAE, only about 15% of the fibrous elements in the white matter were NTPDase2 single-labeled elements, while the others were either NTPDase2/nestin double-labeled or nestin single-labeled (Figure 5R).

**Figure 6:**
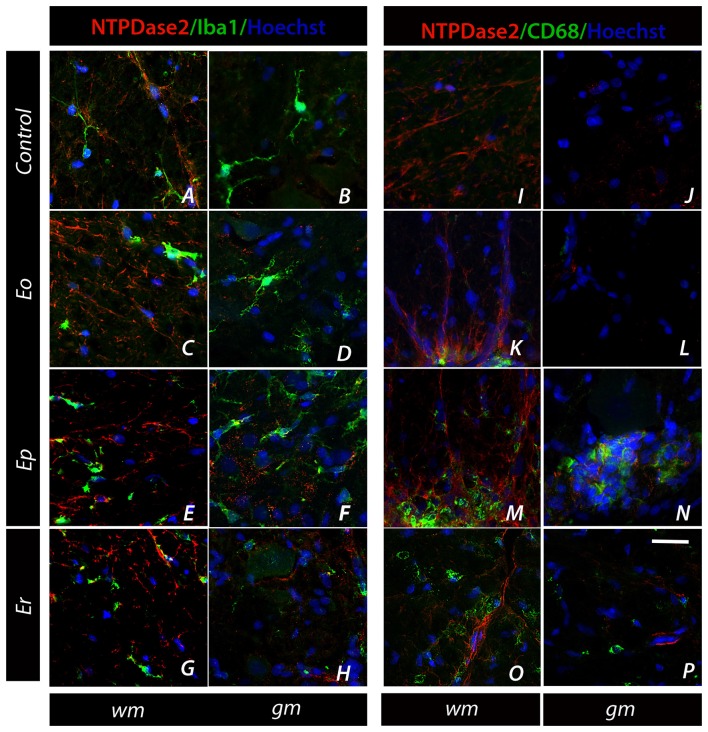
Association of NTPDase2 with markers of microglia. **(A–H)** Representative micrographs showing double IF labeling directed to NTPDase2 (*red fluorescence*) and Iba-1 (*green fluorescence*) at spinal cord sections in control and during EAE. **(I–P)** Representative micrographs showing double fluorescence labeling directed to NTPDase2 (*red fluorescence*) and CD68 (*green fluorescence*). Nuclei are counterstained with Hoechst (*blue fluorescence*). The scale bar at **(P)** applicable to all micrographs = 20 μm.

The expression of NTPDase2 was also studied in microglial cells, which were strongly activated during EAE (Figure 6, Supplementary Figure S4). The association was assessed by double IF labeling using anti-Iba1 and anti-CD68 antibodies, which tag different functional states of microglial cells. Although NTPDase2-*ir* was never observed in either quiescent or activated microglia, the latter cells exhibited a spatial association with NTPDase2-*ir* elements during EAE. Namely, at ***Ep***, a number of ovoid Iba1-*ir* cells and CD68^+^ cells were seen (Figures 6E,M), often in the proximity or surrounded by NTPDase2-*ir* elements (Figures 6E–N).

No co-localization was observed between NTPDase2 and cellular markers for premyelinating oligodendrocytes (O4), oligodendrocyte progenitor cells (NG2), mature myelin (MBP) and neurons (NF-H subunit), as determined by double IF (data not shown).

### Expression of NTPDase2 at CD4^+^ T Cells

**Figure 7:**
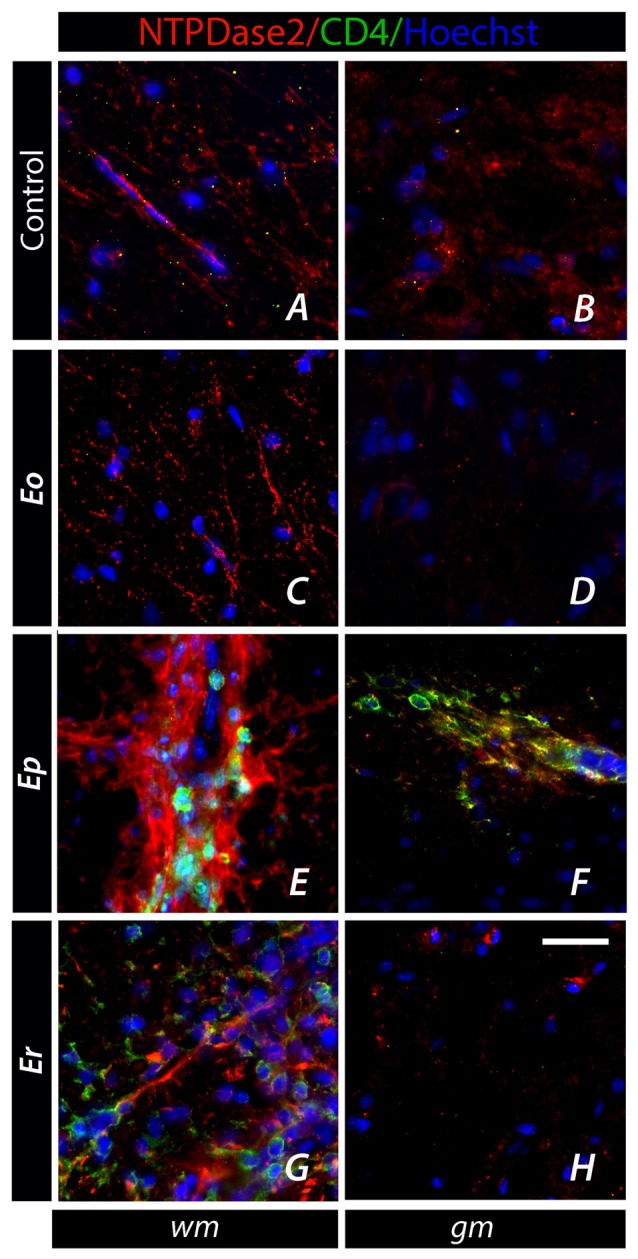
Association of NTPDase2 with CD4^+^ infiltrates in EAE. **(A–H)** Representative micrographs showing cellular infiltrates comprising CD4^+^ and CD4^−^ cells over the course of EAE. CD4^+^ cells (*green fluorescence*), which appear at ***Ep***, were usually surrounded by NTPDase-*ir* elements, whereas colocalization of NTPDase2 at CD4^+^ was never observed. Nuclei were counterstained with Hoechst (*blue fluorescence*). Scale bar at **(H)** applicable to all micrographs = 20 μm.

**Figure 8:**
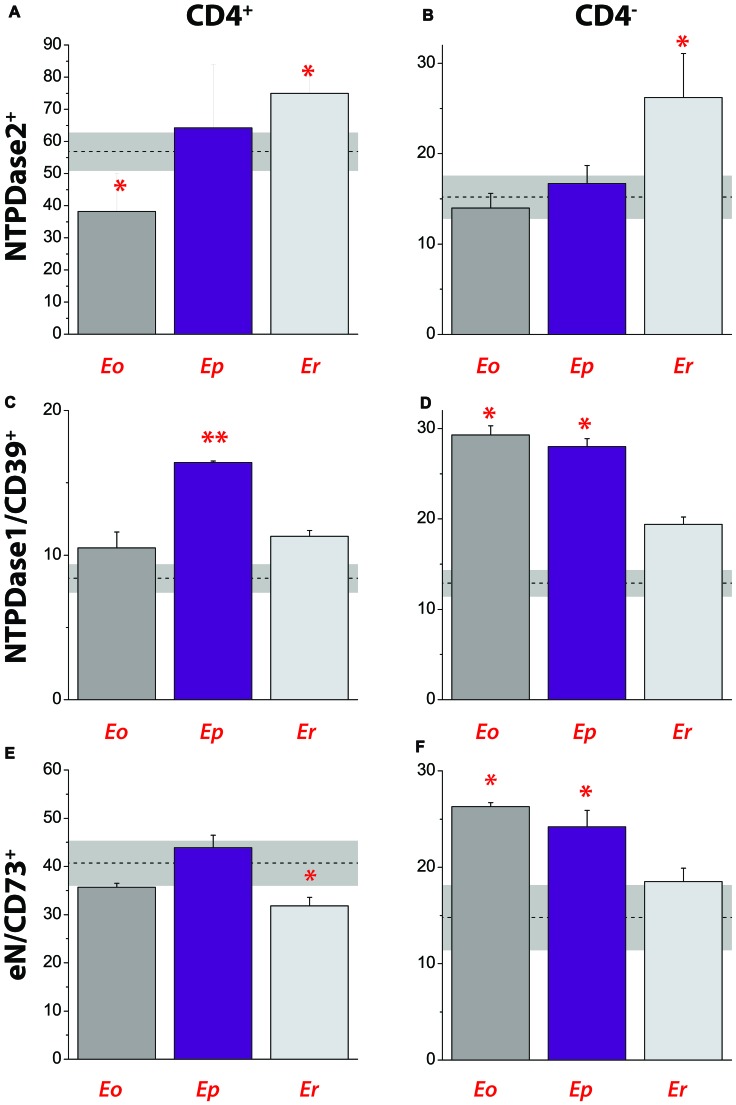
Contribution of NTPDase2^+^, NTPDase1/CD39^+^ and eN/CD73^+^ cells to total cells in draining lymph node during EAE. Contribution of NTPDase2^+^ cells to CD4^+^ **(A)** and CD4^−^ **(B)** cell populations. Contribution of NTPDase1/CD39^+^ cells to CD4^+^ **(C)** and CD4^−^ **(D)** cell populations. Contribution of eN/CD73^+^ cells to CD4^+^ **(E)** and CD4^−^ **(F)** cell populations. Dot lines at each graph represent mean contribution (%) ± SEM (*gray area*) of the target of interest in total T cell population in DLN, determined in control animals. Bars represent mean contribution (% ± SEM) of cells expressing target antigen in total T cell population in DLN, as determined from two separate lymphocyte isolations and fluorescence-activated cell sorting. Significance inside the graph: *p < 0.05; **p < 0.001.

Given the fact that EAE is mediated by self-reactive CD4^+^ T cells entering the spinal cord parenchyma, we explored the surface expression of NTPDase2 at CD4^+^ cells, both at spinal cord infiltrates (Figure 7) and in a draining lymph node (DLN, Figure 8). The cells infiltrated in the spinal cord parenchyma were first seen at ***Ep***, as clusters of CD4^+^ and CD4^−^ cells, ranging from few to several dozens per ×40 field (Figures 7E,F). These CD4^+^ T cells were never observed to express NTPDase2. On closer examination, the infiltrates were often seen in juxtaposition with NTPDase2-*ir* fibrous elements, which appeared to surround or wall off the clusters of the inflammatory cells (Figure 7E). At ***Er***, numerous infiltrates were still seen, particularly in the dorsal white matter (Figure 7G).

The contribution of NTPDase2^+^ cells to total cell population in DLN was analyzed by fluorescence-activated cell sorting, after staining the cells with fluorescence-labeled antibodies directed against CD4 and NTPDase2 (Figures 8A,B). In parallel, we analyzed the contributions of NTPDase1/CD39^+^ (Figures 8C,D) and eN/CD73^+^ cells (Figures 8E,F) in the same lymph node. In control animals, CD4^+^ T cells comprised ~62% of total DLN cell population and contributions of NTPDase2^+^, NTPDase1/CD39^+^, and eN/CD73^+^ cells are presented in Table 4. Among CD4^+^ T cells, more than 50% were NTPDase2^+^ (Figure 8A), and about 40% were eN/CD73^+^ (Figure 8E), while only 10% were NTPDase1/CD39^+^ (Figure 8B). In contrast, the contribution of NTPDase2^+^ cells to CD4^−^ cell population in control animals was much lower (Figure 8B). The onset of disease was associated with a transient decrease in the number of CD4^+^ NTPDase2^+^ cells (Figure 8A) and notable increase in NTPDase1/CD39^+^ (Figure 8D) and eN/CD73 cells (Figure 8F) within CD4^−^ cells. At ***Ep***, a general increase in NTDPase1/CD39^+^ cells (Figures 8C,D) and an increase in eN/CD73^+^ cells within the CD4^−^ cell population (Figure 8F) were observed. At ***Er***, the contribution of NTPDase2^+^ cells increased in both CD4^+^ (Figure 8A) and CD4^−^ cells (Figure 8B), the contribution of eN/CD73^+^ cells in CD4^+^ T cells decreased (Figure 8E), while distribution of NTPDase1/CD39 cells returned to the baseline (Figures 8C,D).

**Table 4:** Contribution of CD4^+^, NTPDase2^+^, NTPDase1/CD39^+^ and eN/CD73^+^ cells to total population of cells in draining lymph node during EAE.

	Control	*Eo*	*Ep*	*Er*
**(% of total cell population)**				
CD4^+^	62.2 ± 1.3	52.1 ± 0.8*	58.8 ± 1.2	53.9 ± 0.8*
NTPDase2^+^	50.7 ± 3.0	38.6 ± 2.9*	54.0 ± 3.2	64.3 ± 5.2
CD39^+^	23.3 ± 1.2	39.8 ± 1.4*	44.4 ± 1.4*	30.7 ± 0.6*
CD73^+^	53.0 ± 7.45	62.1 ± 0.9	68.0 ± 4.3	50.2 ± 2.9

Statistical significance: *p < 0.05.

## Discussion

The growing body of literature supports the view that extracellular purine nucleotides and nucleosides play important roles in neuron-to-glia and glia-to-glia communications, both in health and disease (Fields and Burnstock, 2006; Franke et al., 2012; Burnstock, 2016). Compelling evidence support the critical role of P2X7-, A1- and A2A-mediated signaling in the pathophysiology of EAE (for review, see Cieślak et al., 2011; Safarzadeh et al., 2016), while the involvement of ADP-sensitive P2Y_12_ receptor is recently implicated as well (Qin et al., 2017; Zhang et al., 2017). The goal of the present study was to complement existing knowledge by studying the expression of NTPDase2 and ADP-sensitive P2 receptors in the spinal cord over the course of EAE since their contribution to the pathophysiological process is largely unknown.

Our study confirms that NTPDase2 exhibits a selective localization in the CNS (Braun et al., 2004; Shukla et al., 2005; Mishra et al., 2006; Gampe et al., 2015), in contrast to general expression by astrocytes *in vitro*, where it represents the major ectonucleotidase (Wink et al., 2006; Brisevac et al., 2015). In the rat spinal cord, NTPDase2 is restricted to the white matter, where it resides in most of the GFAP-*ir* fibrous astrocytes, while typical protoplasmic astrocytes, neurons, microglia, oligodendrocytes and NG2^+^ cells are essentially devoid of NTPDase2. The NTPDase2-containing fibrous astrocytes are elongated and similar in appearance to laminar astrocytes which form sheets around myelinated fiber bundles (Braun et al., 2003; Shukla et al., 2005; Gampe et al., 2012). Among these NTPDase2-*ir* elements in the spinal cord, roughly 20% co-express neural stem cell marker nestin, indicating that they may belong to neural stem cells (Shukla et al., 2005; Mishra et al., 2006; Gampe et al., 2015), scattered in the spinal white matter (Wei et al., 2002). Therefore, NTPDase2 is present in the rat spinal cord, in a population of GFAP-*ir* fibrous astrocytes, some of which may belong to adult progenitor cells.

The present study discloses a novel finding that the protein levels of NTPDase2 in the spinal cord alter during EAE in a disease-phase specific manner, wherein the decrease in the membrane expression directly correlates with a severity of symptoms. Namely, during the symptomatic phases of EAE, the mRNA and protein expression of NTPDase2 progressively decrease, while the recovery in NTPDase2 gene and protein expression accompanies the recovery from the disease. Counting the number of GFAP single-labeled and GFAP/NTPDase2 double-labeled elements at each phase of the disease revealed that the intensity of NTPDase-*ir*, but not the number of NTPDase2–*ir* elements, change during EAE, indicating that alterations in NTPDase2 expression occur in the same cell subset, i.e., at fibrous astrocytes which typically express NTPDase2 in the normal spinal cord. On the other hand, the contribution of NTPDase2/nestin double-labeled elements increases toward the end of the disease, indicating that the recovery from EAE is associated with the increase in number of progenitor cells in the spinal cord.

While the overall expression of NTPDase2 markedly decreases in lumbosacral part of the spinal cord, the gene and protein expression of NTPDase1/CD39 and eN/CD73 markedly increase at ***Ep*** in comparison to control (Lavrnja et al., 2009, 2015). Of note is that under acute inflammatory conditions *in vitro*, cultured astrocytes also respond by reducing the expression of NTPDase2 (Brisevac et al., 2015). Although only direct *in vivo* measurements of the extracellular nucleotide concentrations would be evidence for a change in the extracellular purine metabolism, the alterations in the expression of the whole enzyme chain for extracellular ATP hydrolysis imply that there are corresponding alterations in the ectonucleotidase activities during EAE. Indeed, a significant increase in ATP, ADP and AMP hydrolyzes has already been demonstrated in the spinal cord tissue during the symptomatic phase of EAE (Lavrnja et al., 2009, 2015), with the kinetic parameters which indicate an increase in CD39/NTPDase1 and reduction in NTPDase2 hydrolyzing activities (Lavrnja et al., 2009). The aforementioned shifts in the expression and activity of ectonucleotidases presumably result in the lower availabilities of ATP and ADP as ligands for the respective P2 receptors. We, indeed, found significantly altered expression of ADP-sensitive P2 receptors, i.e., down-regulation of P2Y_1_ and P2Y_12_ receptor proteins at the onset of EAE, and several-fold increase in the expression of P2Y_12_ and P2Y_13_ receptor proteins at ***Ep*** and/or ***Er***. The results of gene expression analyses also demonstrated consistent down-regulation of P2X purinoceptors genes (this study; Lavrnja et al., 2015) and a shift in the expression of P1 receptor genes, i.e., downregulation of A_1_ and A_2B_, moderate increase in A_2A_ and several-fold induction in A_3_ receptor genes. Although based on transcriptional analyses, our results corroborate essential and complex role(s) of ATP- and adenosine-mediated signaling in the pathogenesis of MS/EAE (for review, see Cieślak et al., 2011; Safarzadeh et al., 2016).

What (patho)physiological implications emerge from these data? Disease-phase specific alterations in NTPDase2 protein expression suggest that there are corresponding alterations in the enzyme activity and the availability of ADP in the extracellular milieu during EAE. These data, together with the altered expression of ADP-sensitive P2 receptors suggest that pathophysiological processes in EAE may be associated with the differential activity of ADP signaling. Among three of the dominant ADP-sensitive P2Y receptors, only P2Y_12_ receptor have been implicated so far in the pathophysiology of MS/EAE. The study of Amadio et al. (2010) reported that the expression of P2Y_12_ receptor protein in the vicinity of demyelinating lesions inversely correlated with the extent of demyelination in MS. The P2Y_12_ receptor exhibits specific cellular localization, being expressed by oligodendrocytes (Sasaki et al., 2003; Amadio et al., 2010) and quiescent microglia (Sasaki et al., 2003), while its down-regulation in ramified microglia reflects the cell transition from quiescent to the activated phenotype. In this respect, the established localization of NTPDase2 at fibrous astrocytes which are wrapping myelinated fibers and its absence from protoplasmic astrocytes, neurons, and microglia may suggest a role of NTPDase2 in ADP-mediated signaling between the axon/oligodendrocyte unit on one side and surveillant microglia on the other. Specifically, in normal conditions, the electrical activity of axons causes the tonic release of ATP, which, after being converted to ADP, activates the P2Y_12_ receptor and sustains quiescent microglial phenotype. Pathological or homeostatic imbalance may induce down-regulation of NTPDase2 and consequent decrease in ADP-dependent P2Y_12_ receptor activation at oligodendrocyte/microglia interface. This may act as an alarm signal to promote the transition of quiescent microglia to M2/M1 phenotype and the induction of neuroinflammation. A lack of P2Y_12_ receptor activation may also promote direct or indirect effects on oligodendrocyte survival and axonal loss (Amadio et al., 2010). In addition, a recent article describes the critical role of P2Y_12_ receptor in Th17 differentiation and pathogenesis of EAE (Qin et al., 2017).

Another novel finding presented herein is the significant up-regulation of P2Y_13_ receptor at ***Er***, indicating that the induction of this P2 purinoceptor may be associated with the resolution of neuroinflammation and a recovery from the disease. The suggestions are corroborated by findings that the activation of P2Y_13_ receptor induces main pro-survival pathways in neurons, such as ERK1/2 and PI3K/Akt/GSK3 (Pérez-Sen et al., 2015), which are normally activated by trophic factors, and the activation of the Nrf2/HO-1 axis which protects from excitotoxic damage (Espada et al., 2010). These findings link ADP to neuroprotection and imply that, in contrast to ATP, ADP may promote survival and increased neuronal resistance to different insults (del Puerto et al., 2012). In this sense, activation of the P2Y_13_ receptor may have a pivotal role in challenging conditions during neuroinflammation and therefore, may provide a potential target for the neuroprotective action in MS/EAE.

Given the role of CD4^+^ T cells in the pathophysiology of EAE/MS (McFarland and Martin, 2007) and the fact that these cells significantly contribute to mononuclear tissue infiltrates, the second goal of the present study was to explore the association of NTPDase2 with CD4^+^ cells at distinct phases of EAE. To our knowledge, this is the first report on the occurrence and abundance of CD4^+^NTPDase2^+^ T cells, their infiltration into the spinal cord and temporal changes in EAE. Since our study did not differentiate between subsets of CD4^+^ T cells, i.e., Th1, Th2, Th17, Tfh and Treg (Nakayamada et al., 2012), the findings obtained in our study may support only three broad conclusions: (*a*) the presence of NTPDase2 does not distinguish between the two main T cell subsets, as both CD4^+^ and CD4^−^ cells in DLN expressed NTPDase2. However, it is of note that in physiological conditions, a considerable number of CD4^+^ cells expressed NTPDase2. (*b*) The onset of EAE coincided with a reduction in CD4^+^NTPDase2^+^ cell number at DLN, whereas the resolution went together with the elevation of both CD4^+^NTPDase2^+^ and CD4^−^NTPDase2^+^ cell numbers. The opposite was found for cells expressing NTPDase1/CD39 and eN/CD73, which were slightly increased in number at the onset of EAE and returned to baseline level at the recovery. (*c*) Spinal cord infiltrates, comprising a mixed population of CD4^+^/CD4^−^ cells, never expressed NTPDase2. It is widely accepted that the neurological impairments in EAE are induced by pathogenic CD4^+^ infiltrates, which abundantly express NTPDase1/CD39 and eN/CD73 (Hernandez-Mir and McGeachy, 2017). Given the significant number of CD4^+^NTPDase2^+^ T cells in DLN, it is of note that these cells were never observed within CNS infiltrates. The finding also suggests that CD4^+^ NTPDase2^+^ cells do not enter the CNS parenchyma during EAE and do not contribute to the initiation of EAE.

Beyond these conclusions, a literature survey on regulatory elements and acting transcription factors in the promoter region of a gene encoding NTPDase2 led us to several interesting implications. The promoter region of rat *Entpd2* comprises two IL-6 responsive elements (IL-6 RE; Yu et al., 2008). The interleukin (IL)-6, which functions as Th17 and B cells growth and differentiation factor (Serada et al., 2008), attracts increasing attention in recent years as an important cytokine involved in inflammatory diseases of the CNS, including MS/EAE (Fujimoto et al., 2008; Janssens et al., 2015). Since markedly elevated IL-6 levels are detected in CSF and plasma of MS patients, and since the cytokine acts as a suppressor of *Entpd2* (Yu et al., 2008), it is most likely that the down-regulation of NTPDase2 during EAE is induced by IL-6-mediated suppression of *Entpd2*. Promoter regions of human *ENTPD2* and mouse *Entpd2* contain yet another important immune-related regulatory binding site, GATA-3 binding site (Chadwick and Frischauf, 1997). GATA-3 is a zink-finger transcription factor that directs CD4^+^ cell polarization towards Th2 subtype, by inducing expression of Th2-type cytokines (Zhu et al., 2004; Evans and Jenner, 2013) and by inhibiting expression of Th1-type master cytokine, interferon-γ (Lee et al., 2000). Therefore, since *ENTPD2* is one of the GATA-3 linked genes (Dydensborg et al., 2009), it may be speculated that its expression on T cells characterizes the fully developed Th2 cell phenotype. Since Th1 plays role in induction, while Th2 response prevails in the recovery phase of EAE (Nagelkerken, 1998), a second implication that may emerge from our data is that the reduction of CD4^+^/NTPDase2^+^ cell number during ***Eo*** may reflect prevalence of Th1, while increase in CD4^+^/NTPDase2^+^ cell number may reflect a shift to Th2 response during recovery phase of EAE.

To summarize, the main goal of our study was to complement the existing knowledge on the involvement of purinergic signaling in the sequence of events leading to EAE, by describing alterations in NTPDase2 and ADP-sensitive P2 receptors expression. Our results demonstrate that the onset of EAE is associated with downregulation of gene and protein expression of NTPDase2, P2Y_1_ and P2Y_12_ receptors, while the recovery from EAE is associated with the baseline gene and protein expression of NTPDase2 and P2Y_1_ receptor and strong upregulation of P2Y_12_ and P2Y_13_ receptors in the spinal cord of the affected animals. The findings suggest a possible link between ADP signaling and the pathophysiological processes in EAE. Further studies are needed to unveil the signals that initiate the observed alterations in NTPDase2 expression and embed them in the neuroinflammatory cascade which results in EAE and neuroimmune diseases in general.

## Data Accessibility

The following tools, software and databases were used: Image analyses were conducted using *ImageJ* (http://imagej.nih.gov/ij/download.html; RRID:SCR_003070). Statistical analysis was performed using OriginPro 8.0 Software package (http://www.originlab.com/index.aspx?go=PRODUCTS/Origin; RRID:SCR_014212).

## Author Contributions

All the listed authors made sufficient contributions to the design of the work, acquisition, analyses, and interpretation of the data, participated in drafting the work, gave final approval of the submitted version of the manuscript and agreed to be accountable for all aspects of the work. IL, MJ, DL, NN: conceived and designed; MJ, IBo, IL, DL, IBj, DS, SP: performed experiments; NN, DL, MJ, IL: analyzed the data; NN, DL, IL, MJ, IBj, SP, IBo, DS, JS: contributed to the writing.

## Conflict of Interest Statement

The authors declare that the research was conducted in the absence of any commercial or financial relationships that could be construed as a potential conflict of interest.

## Supplementary Material

FIGURE S1 Western blot calibration curve. **(A)** Twofold serial dilutions of sample proteins were resolved on SDS-PAGE, electrotransferred to Immobilon-P transfer membrane and incubated with rabbit polyclonal primary anti-NTPDase2 antibodies (1:1000 dilution in TBST) and HRP-conjugated secondary antibodies, using ECL substrate. After 1-min film exposure, the support membrane was scanned and the digital image was analyzed using ImageJ. A calibration curve is constructed by plotting the optical density readings against different amounts of sample proteins to construct. The signal was linear accross the range of 9-20 μg of sample proteins. Asterisk denote the sample amount used for quantification of NTPDase2 protein abundance on Western blot. **(B)** Representative support membrane showing serial dilutions of sample proteins probed with the antibodies directed against to NTPDase2 and β-actin.

FIGURE S2 Merged and single-channel immunofluorescence images related to Figures 5A–H. Cross-sections of the lumbosacral part of the spinal cord are stained for NTPDase2 *(red)* and GFAP *(green)* and nuclei are counterstained with Hoechst *(blue)*. The scale bar = 20 μm.

FIGURE S3 Merged and single-channel immunofluorescence images related to Figures 5I–P. Cross-sections of the lumbosacral part of the spinal cord are stained for NTPDase2 *(red fluorescence)* and nestin *(green fluorescence)* and nuclei are counterstained with Hoechst *(blue fluorescence)*. The scale bar = 20 μm.

FIGURE S4 Merged and single-channel immunofluorescence images related to Figures 6I–P. Cross-sections of the lumbosacral part of the spinal cord are stained for NTPDase2 *(red fluorescence)* and CD68 *(green fluorescence)* and nuclei are counterstained with Hoechst *(blue fluorescence)*. The scale bar = 20 μm.
