# Supplementary figures and images for: Down-regulation of NTPDase2 and ADP-sensitive P2 Purinoceptors Correlate with Severity of Symptoms during Experimental Autoimmune Encephalomyelitis
Source: Front Cell Neurosci. 2017 Oct 30;11:333. doi: 10.3389/fncel.2017.00333 (PMC5670145; doi:10.3389/fncel.2017.00333)

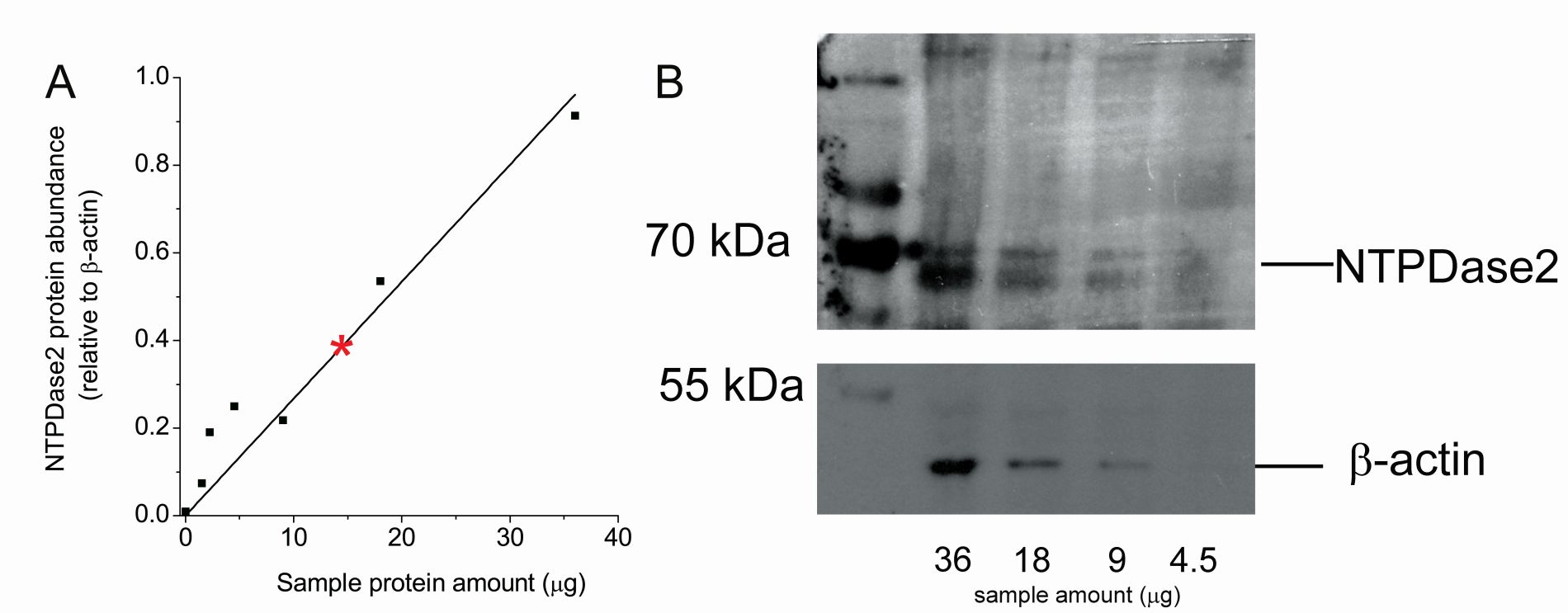

Supplement: FIGURE S1 — Western blot calibration curve. (A) Twofold serial dilutions of sample proteins were resolved on SDS-PAGE, electrotransferred to Immobilon-P transfer membrane and incubated with rabbit polyclonal primary anti-NTPDase2 antibodies (1:1000 dilution in TBST) and HRP-conjugated secondary antibodies, using ECL substrate. After 1-min film exposure, the support membrane was scanned and the digital image was analyzed using ImageJ. A calibration curve is constructed by plotting the optical density readings against different amounts of sample proteins to construct. The signal was linear accross the range of 9-20 μg of sample proteins. Asterisk denote the sample amount used for quantification of NTPDase2 protein abundance on Western blot. (B) Representative support membrane showing serial dilutions of sample proteins probed with the antibodies directed against to NTPDase2 and β-actin. [file Image_1.jpeg]

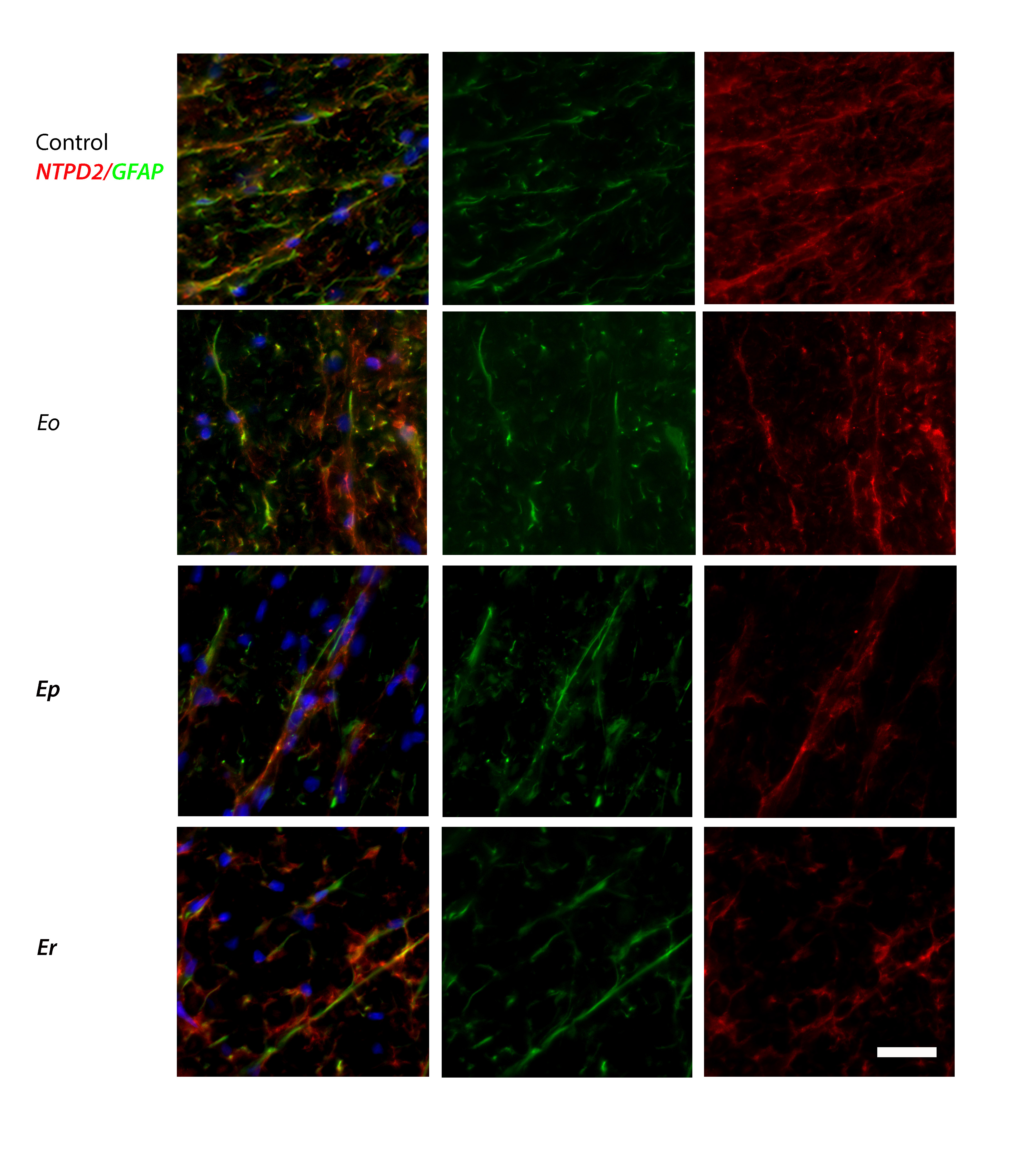

Supplement: FIGURE S2 — Merged and single-channel immunofluorescence images related to Figures 5A–H. Cross-sections of the lumbosacral part of the spinal cord are stained for NTPDase2 (red) and GFAP (green) and nuclei are counterstained with Hoechst (blue). The scale bar = 20 μm. [file Image_2.jpeg]

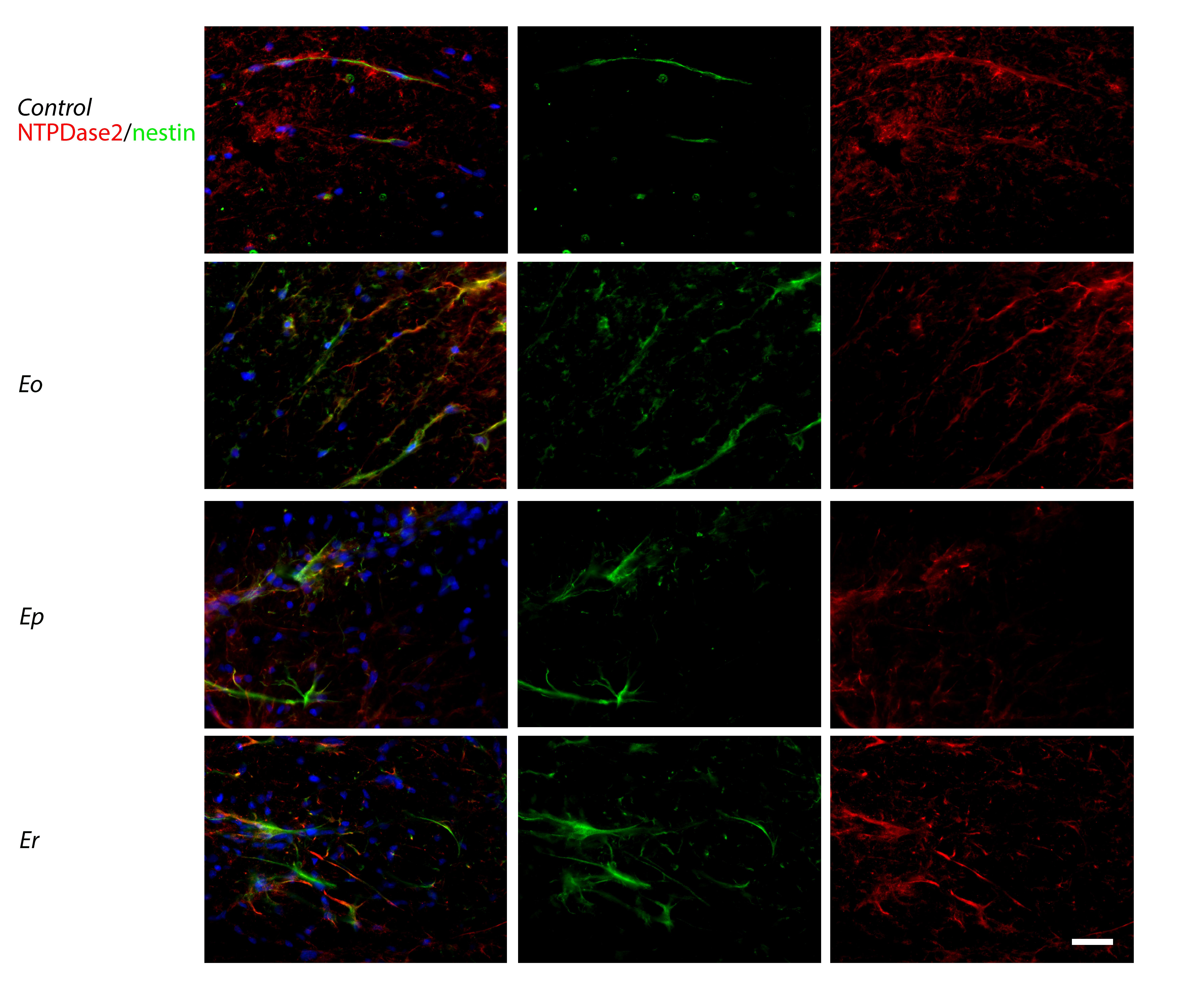

Supplement: FIGURE S3 — Merged and single-channel immunofluorescence images related to Figures 5I–P. Cross-sections of the lumbosacral part of the spinal cord are stained for NTPDase2 (red fluorescence) and nestin (green fluorescence) and nuclei are counterstained with Hoechst (blue fluorescence). The scale bar = 20 μm. [file Image_3.jpeg]

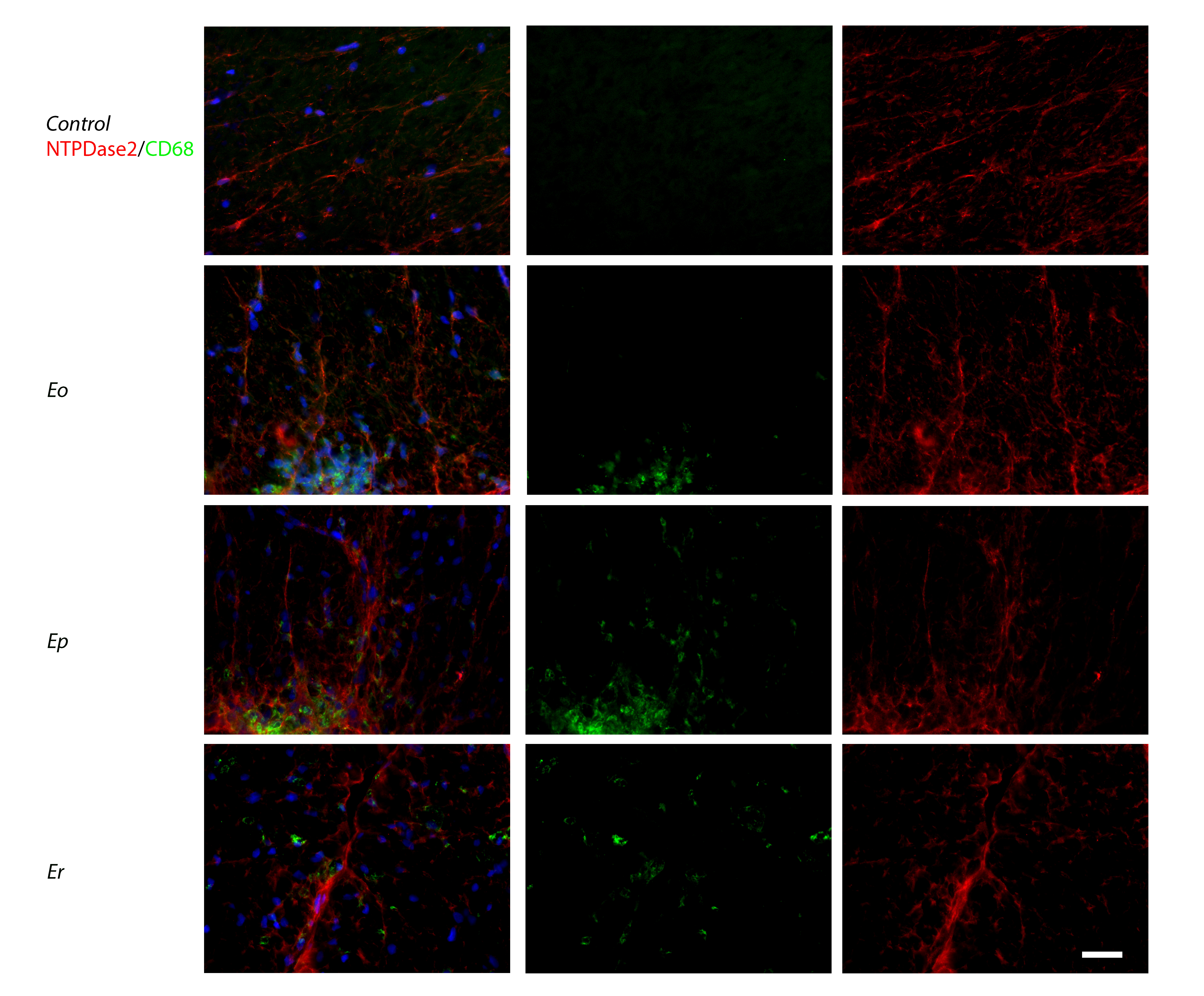

Supplement: FIGURE S4 — Merged and single-channel immunofluorescence images related to Figures 6I–P. Cross-sections of the lumbosacral part of the spinal cord are stained for NTPDase2 (red fluorescence) and CD68 (green fluorescence) and nuclei are counterstained with Hoechst (blue fluorescence). The scale bar = 20 μm. [file Image_4.jpeg]
